# Molecular diversity and function of jasmintides from *Jasminum sambac*

**DOI:** 10.1186/s12870-018-1361-y

**Published:** 2018-07-11

**Authors:** Geeta Kumari, Ka Ho Wong, Aida Serra, Joon Shin, Ho Sup Yoon, Siu Kwan Sze, James P. Tam

**Affiliations:** School of Biological Sciences, Nanyang Technological University, 60 Nanyang Drive, Singapore, 637551 Singapore

**Keywords:** Jasmintides, Cysteine-rich peptides, Antifeedant, Peptide biosynthesis, *Jasminum sambac*

## Abstract

**Background:**

Jasmintides jS1 and jS2 from *Jasminum sambac* were previously identified as a novel family of cysteine-rich peptides (CRPs) with an unusual disulfide connectivity. However, very little else is known about jasmintides, particularly their molecular diversity and functions. Here, we report the discovery and characterization of a novel suite of jasmintides from *J. sambac* using transcriptomic, peptidomic, structural and functional tools.

**Results:**

Transcriptomic analysis of leaves, flowers and roots revealed 14 unique jasmintide precursors, all of which possess a three-domain architecture comprising a signal peptide, a pro-domain and a mature jasmintide domain. Peptidomic analysis, using fractionated mixtures of jasmintides and chemical derivatization of cysteine to pseudolysine, trypsin digestion and MS/MS sequencing, revealed an additional 86 jasmintides, some of which were post-translationally modified. NMR analysis showed that jasmintide jS3 has three anti-parallel β-strands with a three-disulfide connectivity of CysI−CysV, CysII−CysIV and CysIII−CysVI, which is similar to jasmintide jS1. Jasmintide jS3 was able to withstand thermal, acidic and enzymatic degradation and, importantly, exhibited antifeedant activity against mealworm *Tenebrio molitor*.

**Conclusion:**

Together, this study expands the existing library of jasmintides and furthers our understanding of the molecular diversity and cystine framework of CRPs as scaffolds and tools for engineering peptides targeting pests.

**Electronic supplementary material:**

The online version of this article (10.1186/s12870-018-1361-y) contains supplementary material, which is available to authorized users.

## Background

*Jasminum sambac,* which belongs to the Oleaceae family, is a small shrub with fragrant white flowers native to India. Jasmine flowers, regarded as the “queen of flowers”, have traditionally been used for relieving symptoms caused by fever, sunburn, stomach ulcers and anxiety [1]. Its leaves are known to exhibit anti-inflammatory and analgesic properties in rat models [2]. A diverse array of secondary metabolites has been isolated from the flowers and leaves of *J. sambac*, including flavonoids, polyphenols and glycosides [3].

Plants have been a rich source of bioactive compounds and inspiring structures for drug design and discovery [4]. Historically, a major focus of plant-based medicinal research has been directed towards small-molecule metabolites. Recently, peptides have attracted interest as potential therapeutic leads from plants [5–16]. Peptides, however, are generally susceptible to proteolytic degradation, leading to poor metabolic stability or oral bioavailability [6, 9, 11]. In contrast, cysteine-rich peptides (CRPs), particularly those constrained by three to five disulfide bonds, are known to overcome this limitation [6, 9, 14–16]. Consequently, our laboratory has focused on this superfamily of metabolically stable plant-derived peptides, particularly CRPs with molecular weights of 2 to 6 kDa. CRPs in this chemical space generally contain a well-defined tertiary structure due to the cross-embraced multiple disulfide bonds that provide them unprecedented stability against thermal and proteolytic degradation [14–17]. Of particular interest are plant CRPs, which also display a wide range of biological and pharmacological activities [4, 13–16]. As potential therapeutics, plant CRPs with a larger molecular size than small-molecule metabolites have the advantages of increased on-target specificity and decreased off-target toxicity. An additional appeal in discovering novel plant CRPs is their highly stable and evolutionarily conserved structures, which are known to serve as scaffolds that are useful for grafting labile bioactive peptides to increase their metabolic stability in drug development [14–16].

CRPs are classified into different families according to the number of cysteine residues and their disulfide connectivity [4]. Cystine knot peptides, well represented by knottins and hevein-like peptides, are the most commonly encountered six-cysteine CRPs (6C-CRPs) with a knotted disulfide connectivity between CysI−CysIV, CysII−CysV, and CysIII−CysVI [18]. A second family is represented by thionins, comprising a symmetric disulfide pattern or an onion-like topology between CysI−CysVI, CysII−CysV and CysIII−CysIV [19]. Recently, our laboratory reported the discovery of three novel patterns of disulfide threads, including jasmintides, β-ginkgotides and lybatides [7, 13, 16]. The (*Jasminum sambac* peptides) jasmintides, which contain disulfide connectivity between CysI−CysV, CysII−CysIV and CysIII−CysVI, are 6C-CRPs with a 27 amino acid length and a molecular weight of 3.1 kDa, isolated from *J. sambac* leaves [7]. The β-ginkgotides, which contain disulfide connectivity between CysI−CysIV, CysII−CysVI and CysIII−CysV, are hyperdisulfide-constrained peptides with a 20 amino acid length and a molecular weight of 2 to 3 kDa, isolated from *Ginkgo biloba* nuts [16]. The lybatides, which contain disulfide connectivity between CysI−CysVI, CysII−CysVIII, CysIII−CysVII and CysIV−CysV, are cystine-stapled helical peptides with a 33 amino acid length and a molecular weight of 3.6 kDa, isolated from *Lycium barbarum* root bark [13]. Thus far, only two jasmintides (jS1 and jS2) have been reported [7], and little is known about their molecular diversity, precursor sequences, distribution and functions.

Here, we report the molecular diversity, structure and function of jasmintides. We identified 14 non-redundant jasmintide precursors at transcriptomic level which encoding jasmintides jS1-jS15 and 86 novel jasmintides at peptidomic level. NMR structural determination showed that jasmintide jS3 is composed of three anti-parallel sheets constrained by three disulfide bonds with a connectivity similar to that of the previously reported jasmintide jS1 [7]. We also showed that jS3 displays a potent antifeedant effect on *Tenebrio molitor* mealworms*.* Our results expand the library of jasmintides and demonstrate that jasmintides are much more diverse than we previously thought. The highly stable jasmintide structure can potentially be used as a scaffold for grafting bioactive peptides.

## Results

### Isolation of jasmintides jS3 and jS4

**Fig. 1:**
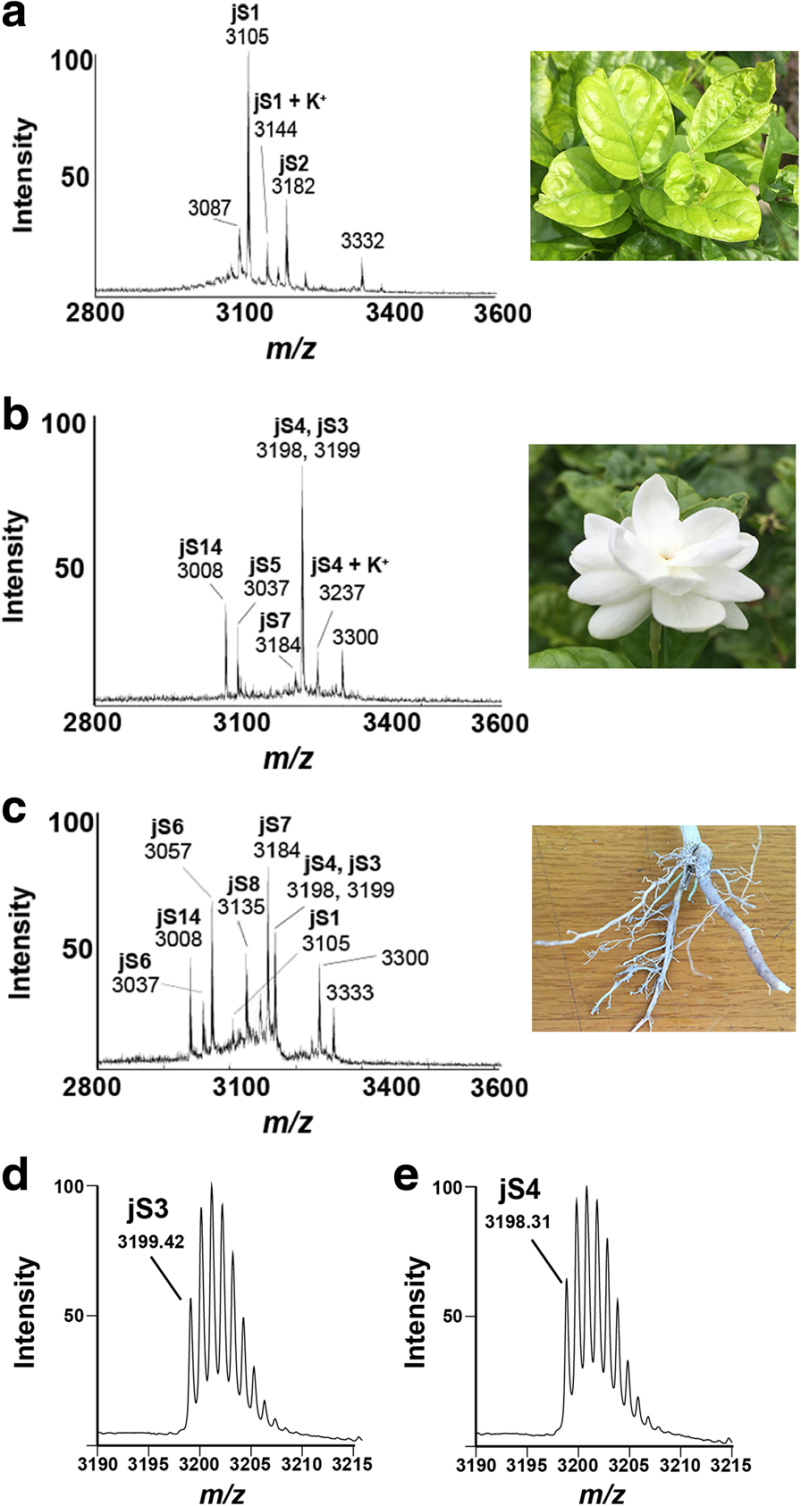
Peptidomic analyses of fresh *J sambac*. MALDI-TOF MS profiles of (**a**) leaves, (**b**) flowers and (**c**) roots of *J sambac*. Zoomed MALDI-TOF MS spectra of purified (**d**) jS3 and (**e**) jS4 with monoisotopic peaks at 3199.42 and 3199.31 Da, respectively

Fresh tissues from the roots, flowers and leaves of *J. sambac* were extracted using 50% (*v*/v) ethanol, purified with C_18_ Ziptip and profiled by MALDI-MS. Distinct MS profiles were observed from different parts of *J. sambac* extracts (Fig. 1). A cluster of peaks was observed between 3000 and 3400 Da of *J. sambac* flowers. To further characterize these compounds, a scale-up extraction of jasmintides was performed using fresh flowers of *J. sambac*, which was then fractionated by reversed-phase flash chromatography and purified by repeated reversed-phase and strong cation-exchange high-performance liquid chromatography. Jasmintides jS3 and jS4, with relative monoisotopic molecular masses [M + H]^+^ of 3199.42 and 3198.31 Da, were isolated, yielding approximately 40 and 20 mg per kg of fresh material, respectively. Jasmintide jS3 was chosen as the representative for further characterization due to its higher abundance than jS4.

### Solution NMR structure of jasmintide jS3

**Fig. 2:**
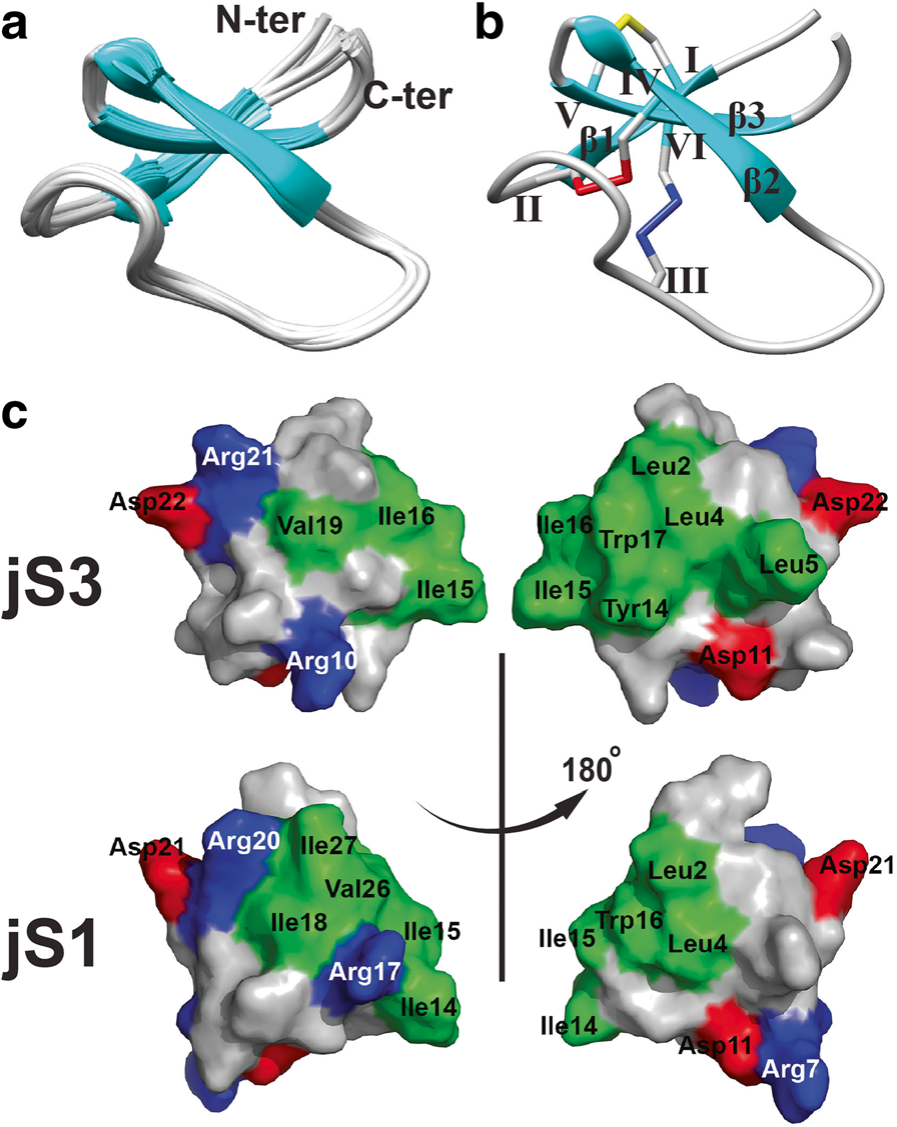
Solution NMR structures of jS3. **a** Superposition of the 20 lowest energy ensembles of jS3. **b** The ribbon NMR representation of jS3, which consists of three anti-parallel β-strands and a disulfide connectivity of CysI−CysV (yellow), CysII−CysIV (red) and CysIII−CysVI (blue). **c** The surface topology comparison between jS3 and jS1. Distribution of electrostatic charges was represented by blue (positively charged), red (negatively charged) and green (hydrophobic residues such as Val, Trp, Ile and Leu)

Two-dimensional nuclear Overhauser effect spectroscopy (NOESY) and total correlation spectroscopy (TOCSY) spectra of jasmintide jS3 with ~ 98% proton resonance were unambiguously assigned. Additional file 1: Table S1 and Additional file 2: Figure S1 illustrate the complete amide proton assignment of the NOESY spectrum. The solution structure of jS3 was determined based on a total of 503 NMR-derived distance restraints and 23 dihedral angle restraints (Table 1). PROCHECK analysis indicated that all residues were distributed in the allowed region of the Ramachandran map. Figure 2a shows the 20 ensembles of jS3 with root-mean-square deviation (RMSD) values of 0.29 Å for backbone atoms and 1.00 Å for all heavy atoms. On the basis of the strong dαN (i, i + 1) NOE connectivity, coupling constants measured by DQF-COSY and 1H-1D experiments, and hydrogen bond patterns determined by amide-hydrogen exchange experiments, jS3 was determined to be composed of a compact disulfide-constrained structure. The overall structure of jS3 consisted of three short antiparallel β-strands (β1: C3 − L5, β2: T18 − R21, and β3: C24 − N26) and two loop segments (L1: C6 − W17 between β1 and β2 and L2: D22 − G23 between β2 and β3), as shown in Fig. 2b and Additional file 3: Figure S2. The disulfide connectivity of jS3 was confirmed by dββ (i, j) and dαβ (i, j) NOE cross-peaks, as illustrated in Additional file 4: Figure S3. NOE signals between Hβ − Hβ from Cys3/Cys24 and Cys6/Cys20 were observed, which corresponded to the CysI−CysV and CysII−CysIV cysteine pairs, respectively. No NOE cross-peak was observed for Cys12/Cys25 due to spectral overlap. However, several long-range NOE correlations were observed between the HN/Hα atoms of Cys3 and Hα/Hβ of Cys23, and therefore, the disulfide connectivity of jS3 was concluded to be arranged as CysI−CysV, CysII−CysIV and CysIII−CysVI, which agrees with the disulfide arrangement determined by partial alkylation reported for jasmintide jS1 [7]. Figure 2c displays the surface topology of jS3. The positively charged residues (Arg10 and Arg21) and negatively charged residues (Asp11 and Asp22) are displayed sporadically, whereas the hydrophobic residues (Tyr14, Ile15, Ile16, and Trp17) in the loop between β-strand 1 and β-strand 2 formed a continuous hydrophobic surface combined with the N-terminal hydrophobic residues (Leu2, Leu4, and Leu5).

**Table 1:** Structural statistics of NMR structures of jasmintide jS3

Number of NOE constraints	
All	503
Intra residues |i-j| = 0	139
Sequential, |i-j| = 1	141
Medium-range, 1 < |i-j| < 5	48
Long-range, |i-j| > =5	175
Number of hydrogen bond constraints	14
Number of dihedral angle constraints	23
Number of constraint violations (> 0.5 Å)	0
Number of angle violations (> 5°)	0
Energies (kcal/mol)	
E_NOE_	0.74 ± 0.33
E_cdih_	0.02 ± 0.01
E_bond_ + E_angle_ + E_improper_	18.53 ± 0.78
E_VDW_	7.27 ± 1.20
RMS Deviation of the structural segment(Cys3-Ser28) for final 20 structures to REM structure	
Backbone(N,C*α*,C′)	0.29 ± 0.06 Å
Heavy atoms	1.00 ± 0.11 Å

### Thermal and enzymatic stability of jasmintide

**Fig. 3:**
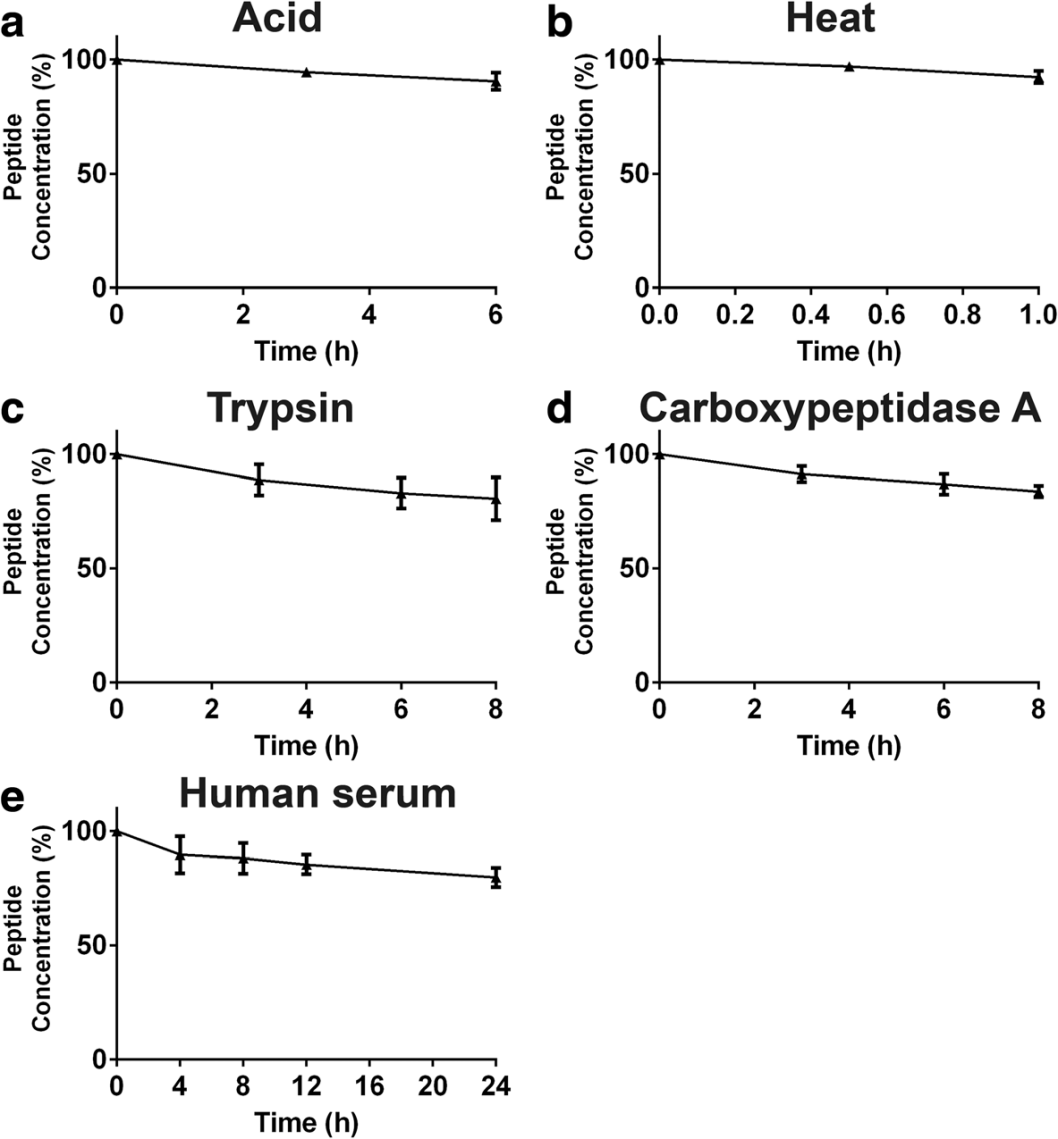
Stability of jS3 against various simulated conditions. **a** Acidic stability was performed at 37 °C and pH 2.0 for six hours. **b** Thermal stability was performed at 100 °C for one hour. **c** Trypsin and (**d**) carboxypeptidase A stability assays were performed at 37 °C for eight hours. **e** Human serum stability was performed at 37 °C for 24 h. The experiments were performed in triplicated (*n* = 3) and were presented as mean ± standard deviation

To show that jasmintide could be a potentially active component in medicinal plants and a highly stable scaffold for grafting bioactive peptides, we performed stability assays mimicking the decoction process and digestive environment (Fig. 3). Chromatographic and mass spectroscopic data revealed that jS3 was resistant in boiling water for 1 h and under acidic conditions (pH 2.0) for up to 6 h with > 92 and > 90% of jasmintide remaining, respectively. Jasmintide jS3 was resistant to the endopeptidase trypsin and the exopeptidase carboxypeptidase A for up to 6 h with > 80% remaining intact. Furthermore, more than 79% of jS3 remained intact after incubation with human serum for up to 24 h at 37 °C.

### Biosynthesis of jasmintides

**Fig. 4:**
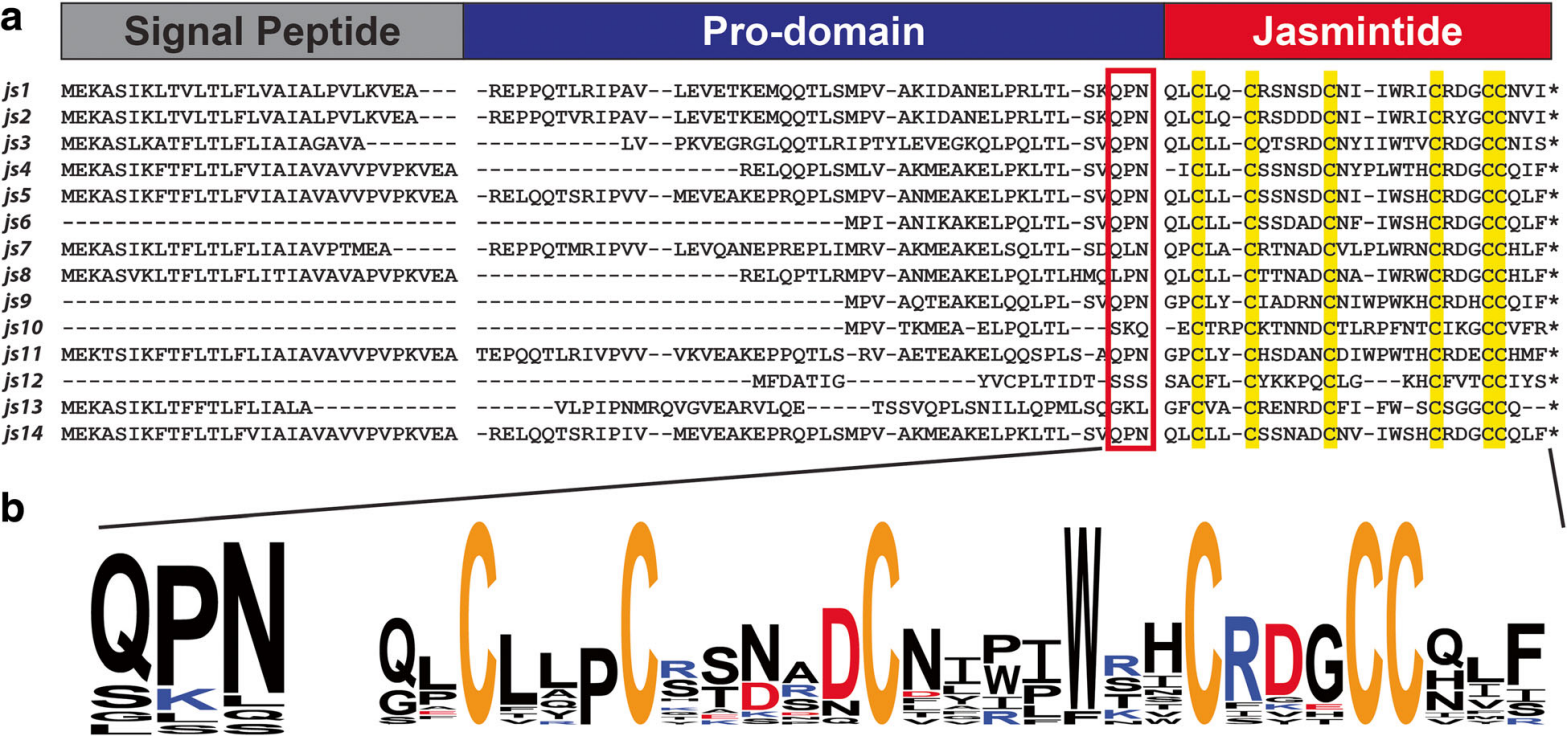
Gene alignment and biosynthetic pathway of jasmintides. **a** The jasmintide precursors consist of an endoplasmic reticulum signal sequence, followed by a prodomain and a jasmintide domain. The asterisks denote the positions of the stop codon. The cleavage sites of the signal peptide are predicted by SignalP 4.1. Conserved cysteine residues in the jasmintide domain are shaded with yellow. **b** The proposed pro-domain cleavage site and jasmintide mature domain were summarized as Weblogo. Gene accession number: js1 (KT 438682.1), js2 (KT 438682.1) and js3 to js14 (KT 472798.1 to KT472809.1)

Total RNA extraction was performed from the roots, flowers and leaves of *J. sambac*, and RNA was sequenced using an Illumina Hiseq 2000. A total of 284.2 million clean reads were generated, with 41.7% GC content and an average length of 829 bp. The raw cDNA reads were assembled by Trinity into 296,026 contigs with a minimum length of 201 nucleotides. A tBLASTn search using the reported jasmintides jS1 and jS2 as queries led to the identification of 14 unique precursor sequences (Fig. 4a). In addition, we found two precursors, *js2* and *js14*, which were identical to those previously reported by our laboratory via transcriptomic analysis [7]. All 14 full-length precursors, each about 100 amino acids in length, shared a three-domain architecture, consisting of an endoplasmic reticulum signal peptide (27–33 aa), a pro-domain (27–47 aa) and a mature jasmintide domain (25–28 aa). The three-domain precursor architecture has been found in other CRP families such as cystine knot α-amylase inhibitors and carboxypeptidase inhibitors. It differs from the two-domain precursor arrangements of thionins and 6C-hevein-like CRPs, which do not have a pro-domain [5, 6, 9, 14, 16]. In addition, a conserved cleavage site, QXN, was found in 11 out of 14 jasmintide precursors at the C-terminus of the pro-domain. Thus, it is likely that an asparaginyl endopeptidase is involved in the bioprocessing of the jasmintides [9, 20]. The remaining three jasmintide precursors (*js10*, *js12* and *js13*) did not contain a QXN processing motif (X represents any amino acid), suggesting that a different processing enzyme could be involved (Fig. 4b). Interestingly, apart from the six highly conserved cysteine residues, some jasmintides were rich in hydrophobic leucine, isoleucine and valine residues.

### Derivatization of cysteine to pseudo-lysine

A mass-spectrometry-driven peptidomic analysis of CRPs using mixtures would be useful to study the molecular diversity of jasmintides. Our laboratory recently developed a one-pot derivatization method to convert cystine into pseudo-lysine, as shown in Fig. 4 [21]. In this one-pot reaction, dithiothreitol (DTT) and 2-bromoethylamine were mixed with fractionated CRPs. DTT reduced cystine to cysteine, and 2-bromoethylame spontaneously cyclized to form an aziridine. Ring opening of aziridine by a cysteinyl thiol formed from the DTT-reduced cystine resulted in S-alkylation, converting cysteine into pseudo-lysine. The S-alkylated mixture was trypsinized, fractionated and analyzed by MS/MS, similar to conventional proteomic analysis. This one-pot approach enables us to analyze a complex mixture of CRPs and minimizes the labor-intensive purification steps [21].

### Peptidomic analysis of jasmintide expression

**Fig. 5:**
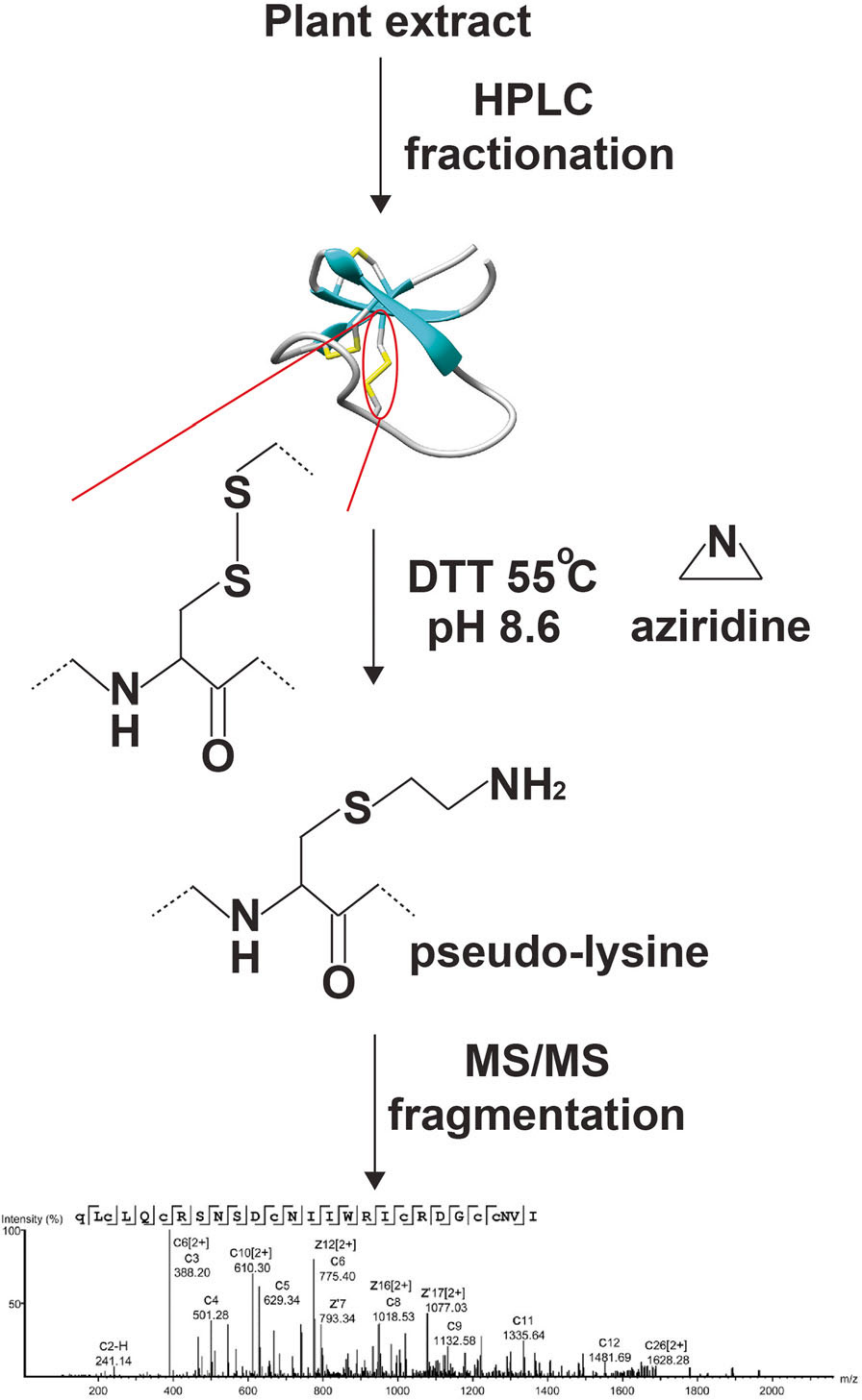
Schematic of peptidomic analysis through the one-pot derivatization of cysteine to pseudo-lysine. Plant extract was fractionated by HPLC and analysed by MALDI-MS. Fractions with peaks between 2000 to 4000 Da were pooled and lyophilized. The dried sample was mixed with dithiothreitol (DTT) and 2-bromoethylamine and incubated at pH 8.6 at 55 °C for 1 h. The reaction mixture was subjected to Orbitrap MS and the fragmentations were matched with the transcriptomic data to confirm the sequences

A mass-spectrometry-driven peptidomic analysis of CRPs using mixtures would be useful to study the molecular diversity of jasmintides and the tissue-specific expression of jasmintides. Individual samples of fresh roots, flowers and leaves of *J. sambac* were extracted with water, fractionated using high-performance liquid chromatography and analyzed using MS as previously described (Fig. 5) [21]. The jasmintide-containing fractions were S-reduced by 30 mM dithiothreitol (DTT) and S-alkylated by 60 mM bromoethylamine at pH 8.6 and 55 °C for one hour. The derivatized mixtures were sequenced by electron-transfer dissociation (ETD) Orbitrap MS (Additional file 5: Figure S4). MS/MS fragmentation data of the alkylated extracts were recorded and queried against the customized transcriptomic database using the PEAKS studio. Identification of unique full or partial sequences with -10LogP values ≥30 was considered valid.

Table 2 summarizes 86 jasmintides identified by MS/MS and nine jasmintide contigs (*js1 − js8* and *js14*) confirmed at the peptidomic level, representing 64.3% coverage of the total number of jasmintide genes present in the *J. sambac* transcriptome. The phylogenetic tree of the 14 jasmintide precursors were illustrated in (Additional file 6: Figure S5). Based on the highly conserved cysteine motif of C-C-C-C-CC and high sequence identity (27.3 to 88.9%) and similarity (36.4 to 92.6%) to the previously reported jS1 (Additional file 7: Table S2), jasmintides jS2 to jS15 were classified as new members of the jasmintide family. Five jasmintide contigs (*jS9 − jS13*) were not detected at the peptidomic level. Our dataset contained 32 full sequences, including jasmintides jS1 and jS2 previously reported by our group [7]. In addition, 12 truncated jasmintide sequences with six cysteine residues and 54 truncated sequences with five or less cysteine residues were identified. A total of 71 sequences were identified in all three parts of *J. sambac*, while three sequences were expressed only in the roots. In addition, eleven sequences were detected in the flowers and leaves of *J. sambac* but were absent in the roots (Table 2). We identified 39 jasmintides with post-translation modifications (PTMs) using peptidomic analysis. They included 34 pyroglutamations at the N-terminus of Gln and six deamidations (five at Asn and one at Gln). Together, we identified 84 novel jasmintides using a combination of transcriptomic and peptidomic approaches.

**Table 2:**
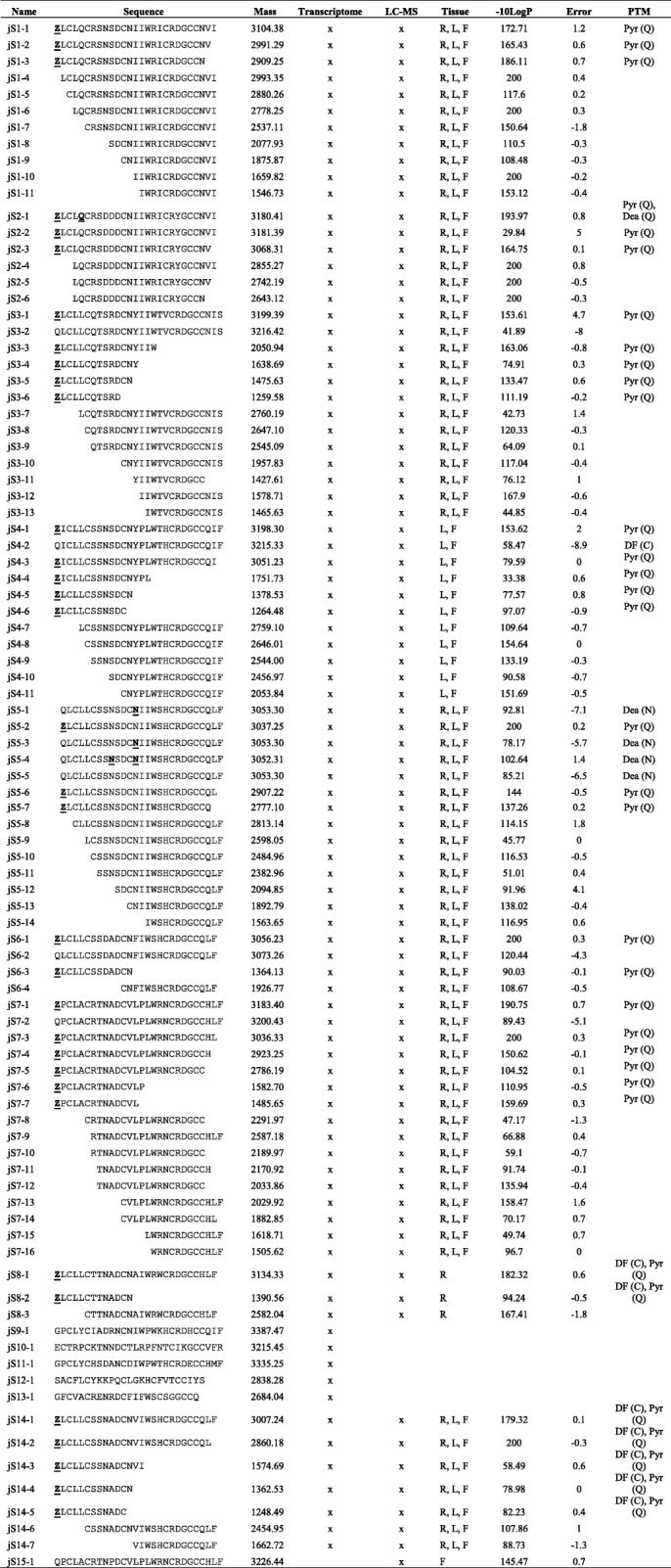
Identification and discovery of jasmintides from transcriptomic and peptidomic approaches

Mass: calculated mass expressed in Dalton. The tissue with the presence of jasmintides was detected by LC-Orbitrap MS/MS, where R, L and F represent roots, leaves and flowers, respectively. A higher -10logP value indicates a more confident sequencing result. Sequences with a -10logP value ≥30 is equivalent to false discovery rate (FDR) < 0.03%. Error represent the error in parts-per million (ppm). *PTM* post-translational modifications, where Pyr and Dea represent pyroglutamation and deamidation, respectively. The bracket represents the post-translational modified amino acid residues. Residues with modifications are bolded and underlined

### Antifeedant activity of jS3 against *Tenebrio molitor* larvae

**Fig. 6:**
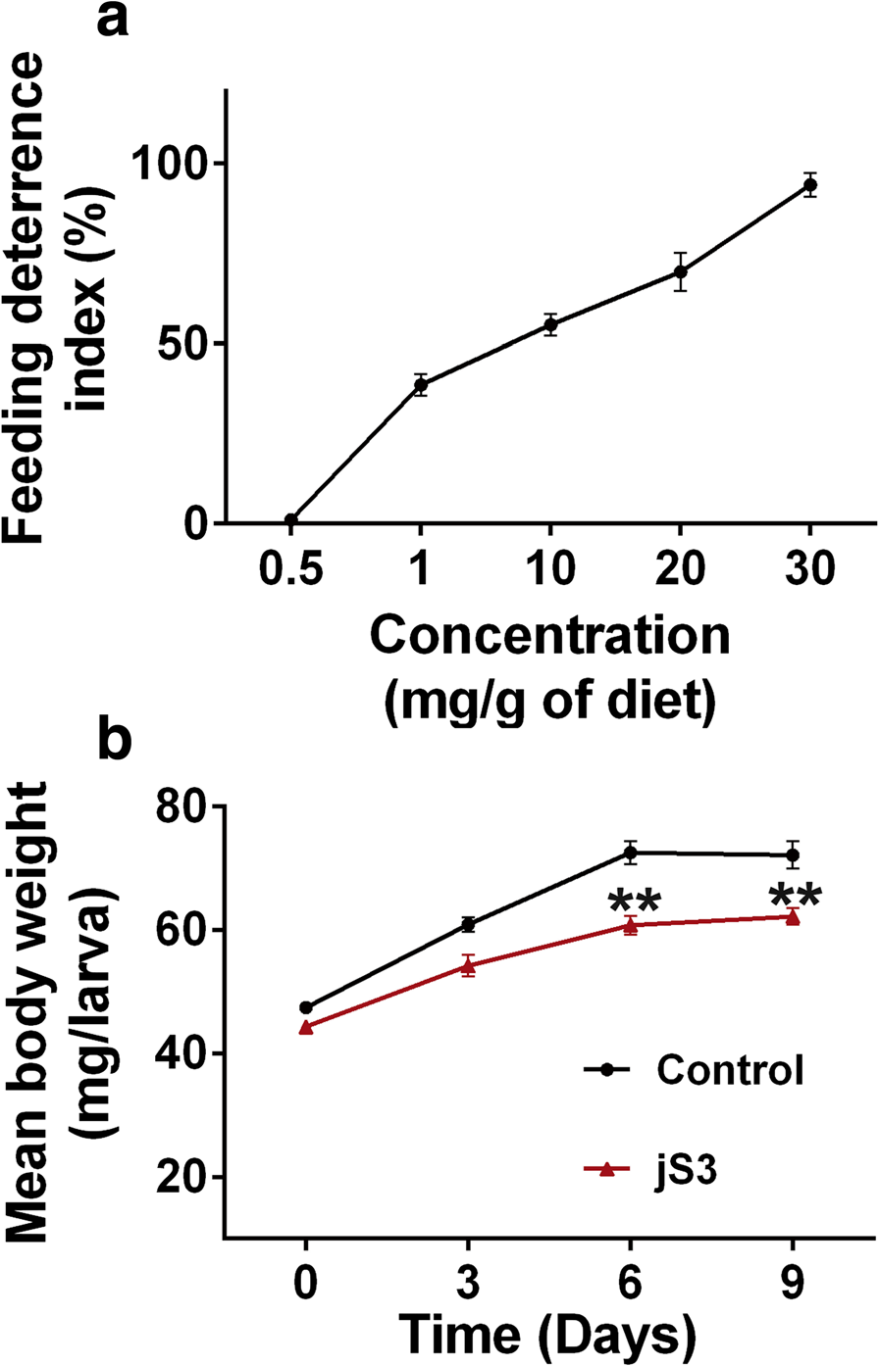
Effect of jasmintide jS3 on *T. molitor* larvae. **a** Feeding deterrence index against increased concentration of jasmintide jS3. **b** Effect of jasmintide jS3 on larvae growth. The body weight of larvae was monitored after every 3 days. All the values were expressed as mean ± standard deviation. Each set of 10 larvae (*n* = 30) were fed with a jS3-containing diet. For comparison of mean with the control, the t-test was used. ** Indicates value with statistically significant (*p* < 0.05) as compared to the control set

To determine the effect of jS3 on *Tenebrio molitor* mealworm larvae, a no-choice assay was performed where the larvae were fed diets containing different amounts of jasmintide (0.5 to 30 mg/g of diet). After 24 h, the remaining food in the control and treatment groups were recorded, and the feeding deterrence index (FDI) was calculated. Figure 6a shows that the FDI increased from 42.9% at 1 mg/g of jasmintide in the diet to the highest value of 93.6% at 30 mg/g of jasmintide in the diet, suggesting a dose-dependent antifeedant effect of jS3 against *T. molitor* larvae. Figure 6b shows the long-term effect of jasmintide on larvae growth, determined using a diet containing 1 mg of jS3 per gram of oat flakes. After a nine-day treatment, the average body weight of the treatment group (*n* = 30) increased from 44.3 to 62.2 mg, which was significantly less than that of the control group without supplementation with jS3, for which the mean body weight increased from 47.4 to 71.2 mg. These results suggested that the jasmintide-containing diet was not preferred by the *T. molitor* larvae; hence, diet consumption in the treatment group was reduced and their mean body weight declined. No fatality was observed during the period of treatment. More importantly, co-incubation of insect Sf9 cells with jasmintide jS3 at concentrations up to 100 μM showed insignificant effects on the change in morphology and mortality rate (Additional file 8: Figure S6).

## Discussion

### Unique cystine framework in jasmintides

Plant CRPs are grouped by their sequence homology and cystine framework. For our purpose, cystine framework is defined as a framework containing both a conserved cysteine motif (one-dimensional) and a conserved disulfide connectivity (two-dimensional). Cysteine spacing refers to the number of amino acids between two cysteine residues and is often referred to as a loop. Thus, jasmintides are a four-looped 6C-CRP family because they contain the cysteine motif of C-C-C-C-CC with a tandem CC motif [7]. Additionally, cystine framework is not equivalent to cysteine motif because CRP families can share the same disulfide connectivity with different cysteine motifs. For example, the knottin family comprises a cystine-knot disulfide connectivity between CysI–CysIV, CysII–CysV and CysIII–CysVI, which is a cystine connectivity found in three CRP families: 6C-hevein-like peptides [5], cysteine-knot α-amylase inhibitors [10, 11] and carboxypeptidase inhibitors [22]. However, the cysteine motifs differ between the families, with a C-C-CC-C-C motif found in cysteine knot α-amylase inhibitors and a C-C-C-C-C-C motif found in carboxypeptidase inhibitors.

The presence of consecutive cysteine residues in a plant 6C-CRP can be used as a clue to identify its family. For example, the CC motif in jasmintides is located at the C-terminus as CysV and CysVI. This C-terminal CC-motif is a mirror image of the cysteine motif found in thionins, whose CC motif is located at the N-terminus as CysI and CysII (CC-C-C-C-C) [4, 7, 19]. On the other hand, the CC motif in the 6C-hevein-like peptides and cystine knot α-amylase inhibitors is located at the middle of the C-C-CC-C-C framework as CysIII and CysIV [10, 14, 15]. In contrast, β-ginkgotides contain two CC motifs with a cystine framework of C-CC-C-CC [16].

Interestingly, the jasmintide-like cysteine motif with a CC motif at the C-terminus has also been found in β-defensin hBD1 isolated from *Homo sapiens* [23] and conotoxin as24a isolated from the murine cone snail *Conus cancellatus* [24] (Additional file 9: Table S3). β-Defensin hBD1 has a similar disulfide connectivity to that of jS3, even though it comprises significantly longer loops 1 and 4 than jasmintides [23]. The disulfide connectivity of as24a has not yet been determined, and it would be of interest to investigate whether it shares similar disulfide connectivity with jasmintides. Molecular diversity also arises in each family with a conserved cystine framework by varying the size and amino acid composition of the displayed loops to diversify their biological function. Compared with jasmintides, hBD1 and as24a are relatively hydrophilic and have been shown to possess antimicrobial and paralytic effects, respectively [23, 24]. This observation suggests that the jasmintide-like framework could have been divergently evolved for several different purposes in plants and animals.

### Unique three-dimensional structure in jasmintides

To determine whether jasmintide jS3 shared a similar peptide fold to other 6C-CRPs, pairwise structure alignment using the TM-align algorithm was performed and is summarized in Additional file 10: Table S4. A TM-score > 0.5 indicates that the two structures shared the same peptide fold, whereas a value < 0.3 represents that they have random structural similarity. Jasmintide jS3 had an average TM-score of 0.21, 0.26, 0.42, 0.38, 0.27 and 0.32 when compared to β-ginkgotide, cystine knot α-amylase inhibitors, carboxypeptidase inhibitors, 6C-hevein-like peptides, thionins and human β-defensin, respectively, whereas its score was 0.56 compared to jS1. Taken together, these results show that jS3 displays a significantly different fold from those of other 6C-CRPs but a high similarity to that of jS1, a result consistent with the notion that they share a similar disulfide connectivity and cysteine spacing [6, 7, 9, 13–16].

### Truncation and PTM increase sequence diversity

The presence of truncated peptides could be contributed by two mechanisms: diversity and turnovers. First, the same gene generates a diverse pool of candidates to respond to various environmental threats for their survival. This is particularly true in small-molecule natural products such as metabolites in which processing enzymes and post-translational modifications as well as degradation provide diversity. Firn and his colleagues proposed the ‘screening model’ to explain how molecular diversity would confer an organism with superior advantages [25]. A pool of diversified molecules “provides a better chance to possess rare components that are useful for changing environments” [21, 25]. Instead of producing a pool of DNA encodes for an array of peptides, plants use one gene to encode multiple peptides via truncation, mutation and other post-translation approaches. In turn, our work shows that such molecular diversity created via biosynthesis and cysteine frameworks can be achieved biosynthetically. Truncation of peptide sequences is a means for diversification and has been studied extensively in cyclotides [21, 26]. In a recent study conducted by Serra et al., a total of 38 cyclotide precursors were identified from *Clitoria ternatea*, which encoded for > 270 cliotides [21]. To show that the presence of truncated peptides are neither methodological artefacts nor generated during sample preparation, the raw plant extract without chemical derivatization was analyzed using LC-MS. The results revealed a wide range of low-abundance shortened peptides with molecular weights ≤3 kDa, suggesting that truncated peptides occurred naturally. In another study, a sum of > 160 cyclotides were identified from the plant extract of *Viola tricolor*, which were encoded by 108 precursor sequences. This “one-gene-multiple-products” approach is more energy conserved than producing multiple genes to adopt the changing environment and has been observed in species other than the plant kingdom. For example, > 100 truncated conotoxins were generated by variable peptide processing from a single gene (MrIA) isolated from the cone snail *Conus marmoreus* [27, 28].

Second, plant peptides and proteins are regularly degraded. Cellular protein degradation is achieved by autophagy of cytosolic complex and organelles through vacuole-based protein degradation and the coordinated action of the proteasome on ubiquitinated proteins in the cytosol [29]. For example, leaf proteins in *Arabidopsis thaliana* rossettes have degradation rates of 0.03–0.04 day^− 1^ and a half-life of 3.5 days [30], whereas the total proteins in *Dactylis glomerata* have degradation rates of 0.12–0.16 day-1 and a half-life of 4–6 days [31]. This is also observed in conotoxins cysteine-rich peptides by Dutertre et al. [27].

Glutaminyl cyclization of the side chain of the N-terminal glutamic acid is one of the most common PTMs found in plant CRPs and has been shown to contribute to stability against enzymatic degradation by exopeptidases such as aminopeptidase I [32]. However, pyroglutamation at the N-terminus could be a spontaneous event. Previous studies demonstrated that deamidation of asparagine is the most likely spontaneous non-enzymatic reaction and is directly related to pH and temperature [33].

### Antifeedant activity of jasmintides

*T. molitor* is not a common pests on *J. sambac*. However, this model has widely been used in determining the insecticidal effect of triterpenes [34], diterprenes [35] and benzoylhydrazine [36], molting disruption effect of long chain n-alkanes [37], and the effect of growth development exerted by pyriproxyfen, a juvenile hormone analog in insect [38]. The use of a similar insect strain generates results that can be compared from various laboratories. The large quantity of larvae required in carrying out various experiments limits the use of common pests. In our study, more than 210 larvae were used. In contrast, *T. molitor* larvae are commercially available in local aquarium store, and hence the results can be easily reproduced in other laboratories. Therefore, instead of a common pest on jasmine, commercially available *T. molitor* larvae were used herein.

It is important to note that the yield of jS3 obtained from the extraction is not the physiological concentration of jasmintide in *J sambac* plant. Since the extraction involved six steps, our estimation is that the physiological concentration would likely be tenfold higher than 40 mg/ kg of fresh material. The antifeedant activity could be explained by three major mechanisms. An olfactory repellent effect is the most common mechanism exerted by volatile aromatic compounds [39]. The second mechanism contributes to the inhibition of digestive enzymes, which disrupts digestion and causes gut discomfort. This mechanism has been observed in 6C-CRPs such as the carboxypeptidase inhibitor from potato *Solanum tuberosum* [40]. The last mechanism relates to true taste deterrence, which stimulates sensory receptors, generates uncomfortable taste and, hence, decreases appetite and food consumption [41]. Certain hydrophobic amino acids have been shown to elicit a bitter taste, including Trp, Tyr, Leu, Ile, Phe and Val [42, 43]. Jasmintide jS3 contains nine such amino acid residues, accounting for 32% of its sequence. Importantly, they are located in close proximity, forming a hydrophobic patch revealed by NMR analysis. As a result, we speculated that the antifeedant effect exerted by jS3 may be related to this hydrophobic patch, which binds to the sensory receptors and evokes a bitter sensation, leading to a decrease in consumption of the jasmintide-containing diet. However, a detailed mechanism of the antifeedant activity exerted by jasmintide remains to be studied.

## Conclusion

In this study, 14 precursor sequences encoding > 80 jasmintides were identified from the roots, flowers and leaves of *J. sambac* using transcriptomic and peptidomic methods. Jasmintides, compact in structure and rich in hydrophobic amino acids such as Leu and Ile, belong to a new family of CRPs with disulfide connectivity that is different from that of knottins and thionins. Jasmintides are resistant to degradation by heat, acid and human serum. Importantly, an in vivo study showed that jasmintide jS3 exhibits an antifeedant effect against *T. molitor* larvae in a dose-dependent manner, suggesting jasmintide jS3 could play a role in host defense. The combination of a compact structure and hyperstability together with in vivo antifeedant effects suggests that jasminitides could be a useful template for developing bioactive peptides. In addition, our results showed that molecular diversity exists on several levels, including amino acid composition, cystine framework, disulfide connectivity and variable peptide processing. Such molecular diversity could be exploited for protein engineering to design feeding deterrents to prevent crop damage.

## Methods

### General materials

*J. sambac* was cultivated at the community herb garden in Nanyang Technological University, Singapore (1° 21′ 22.878″ N, 103° 41′ 23.388″ E). It was authenticated by Mr. Paul Leong from the Singapore Botanic Garden, Singapore, and a voucher specimen was stored at the Singapore Botanic Garden Herbarium Singapore with accession number SING 2015–202. NMR experiments were performed on a 600 or 700 MHz Bruker Avance spectrometer equipped with a 5-mm triple-resonance cryogenic probe head and a z-axis gradient coil at 25 °C. Mass spectrometry analysis was monitored using a MALDI-TOF/TOF ABI 4800 (Applied Biosystems, Framingham, MA, USA). Data acquisition was performed in reflection mode using a mass range of 1000–6000 Da and a focusing mass of 3000 Da. A saturated solution of α-cyano-4-hydroxycinnamic acid prepared in 75% acetonitrile and 0.1% trifluoroacetic acid was used as the MALDI matrix. Desalting of samples was performed as needed using C_18_ ZipTip. HPLC and ultra-performance liquid chromatography (UPLC) were performed on a Shimadzu system. Grace Vydac C_18_ columns (particle size, 5 μm; pore size, 300 Å; Hesperia, CA, USA) with dimensions of 250 × 22 mm, 250 × 10 mm, and 250 × 4.6 mm were used for preparative, semi-preparative, and analytical RP-HPLC, respectively. A PolyLC polysulfoethyl A column (250 × 4.6 mm) was used for SCX-HPLC. LC-MS/MS was performed in an Orbitrap Elite mass spectrometer (Thermo Scientific Inc., Bremen, Germany) coupled with a Dionex UltiMate 3000 UHPLC system (Thermo Scientific Inc., Bremen, Germany) using an Acclaim PepMap RSL column (75 μm × 15 cm; 2 μm particles; Thermo Scientific Inc., Bremen, Germany). All chemical reagents used in this study were of analytical grade and were purchased from Sigma-Aldrich (MO, USA) unless otherwise stated.

### RNA extraction, sequencing and assembly

Total RNA was extracted from the fresh leaves, flowers and roots of *J. sambac* by Trizol according to the manufacturer’s protocol (Life Technologies, California, USA). Sequencing and de novo assembly were performed by Macrogen Inn (Seoul, Korea). Poly-A mRNA was isolated from total RNA using magnetic oligo (dT) beads. Following purification, the mRNA was fragmented into small pieces using divalent cations under elevated temperature. The cleaved RNA fragments were copied into first-strand cDNA using reverse transcriptase and random primers. This was followed by second-strand cDNA synthesis using DNA Polymerase I and RNase H. These cDNA fragments then went through an end-repair process, the addition of a single ‘A’ base, and ligation of the adapters. The products were then purified and enriched by PCR to create the final cDNA library. The cDNA library was examined using an Agilent Bioanalyzer High Sensitivity DNA chip to assess the library quality. Finally, the library was sequenced using an Illumina HiSeq™ 2000 and assembled using Trinity [44].

### Transcriptome analysis of jasmintide precursor sequences

The identification of jasmintide sequences was performed from the raw data using tBLASTn with jasmintides jS1 and jS2 as the query. The e-value cut-off was set at 0.01. The hits were then translated into their respective amino acid sequences via EMBOSS Transeq [45]. Sequences with redundancy and absence of a stop codon were removed. The cleavage sites of precursor sequences were predicted by SignalP 4.1. The jasmintide precursors were aligned using Clustal Omega and visualized by BioEdit. Novel precursor sequences are available in the GenBank database under the accession numbers KY472798.1 to KY472809.1.

### Characterization of jasmintides by mass spectrometry

Fresh *J. sambac* leaves, flowers and roots (800 mg) were washed and individually blended with 50% ethanol (1:3 *w*/*v*). After centrifugation at 1000 rpm for 10 min, the supernatant was collected, diluted to 20% (*v*/v) ethanol and injected into SCX-HPLC. Peaks with intensities > 100 absorption units were pooled and desalted using a C_18_ Sep-Pack. The eluent was concentrated to 200 μL and mixed with 30 mM DTT and 60 mM bromoethylamine in 0.2 M Tris buffer (pH 8.6). The mixture was incubated at 55 °C for 1 h. Subsequently, the alkylated jasmintides were analyzed by HPLC tandem mass spectrometry as previously described, with minor modifications [21]. LC-MS/MS analyses were performed with an Orbitrap Elite mass spectrometer (Thermo Scientific Inc., Bremen, Germany) coupled with a Dionex UltiMate 3000 UHPLC system (Thermo Scientific Inc., Bremen, Germany). Separation of peptides was conducted using a reverse-phase Acclaim PepMap RSL column (75 μm ID × 15 cm, 2 μm particles, Thermo Scientific). The mobile phase was 0.1% formic acid (FA) as eluent A and 90% ACN 0.1% FA as eluent B, with a flow rate of 0.3 μL/min. A 60-min gradient was used for the elution as follows: 3% B for 1 min, 3–35% B over 47 min, 35–50% B over 4 min, 50–80% B over 6 s, and 80% for 78 s and then reverted to the initial state over 6 s and maintained for 6.5 min. Samples were sprayed with a Michrom Thermo Captive Spray nanoelectrospray ion source (Bruker-Michrom Inc., Auburn, CA, USA) set at a source voltage of 1.5 kV and a capillary temperature of 250 °C. Data were collected using Xcalibur 2.2 software (Thermo Scientific Inc., Bremen, Germany) in positive ion mode alternating between full scan-MS (350–1600 *m/z*, 60.000 resolution at 400 *m/z*, 1 microscan per spectrum) and MS/MS (150–2000 *m/z*, 30.000 resolution at 400 *m/z*, 1 microscan averaged per spectrum). High-energy collisional dissociation fragmentation was performed for the 3 most intense ions with a 500-count threshold using 27, 30 and 34% normalized collision energy per spectrum and a 3-Da isolation window. Automatic gain control for full scan-MS and full scan-MS/MS was set to 1 × 10^6^ ions.

The database search and post-translational modification analysis were performed in PEAKS studio (version 7.0, Bioinformatics Solutions, Waterloo, Canada) using 10 ppm MS and 0.05 Da MS/MS tolerances. The PEAKS PTM function [46] was used to search our data for > 650 PTMs and mutations listed in the Unimod database [47]. The SPIDER algorithm was employed to identify peptide mutations and homology [48]. All identified sequences were validated manually.

### Isolation of jasmintides from *J. sambac* flowers

Fresh *J. sambac* flowers (1 kg) were homogenized and extracted with 5 L of 50% (*v*/v) ethanol. After centrifugation (8500 g, 10 min, 4 °C), the supernatant was filtered, diluted to 20% (v/v) ethanol and purified using a C_18_ flash column (Grace Davison, Columbia, MD, USA). The unbound compounds were washed with Milli-Q water and eluted using 1 L of 80% (v/v) ethanol. The eluent was lyophilized, re-dissolved in 200 mL of 20% acetonitrile and purified using several rounds of SCX-HPLC and RP-HPLC, as previously described [9, 10, 49]. Eluents were monitored by MALDI-MS, and fractions with the same molecular weight were pooled and lyophilized. The dried samples were stored at − 20 °C until further analysis.

### NMR spectroscopy

Lyophilized peptides (3 mg) were dissolved in 500 μL of 90% H_2_O/10% D_2_O or 99.99% D_2_O at pH 7.0 in 20 mM Na_3_PO_4_ buffer containing 50 mM NaCl and 0.01% NaN_3_. Homonuclear 2D NOESY experiments were performed with mixing times of 200 and 300 ms. COSY data was recorded in both H_2_O and D_2_O solutions with a mixing time of 78 ms using MLEV17 spin-lock pulses. Vicinal coupling constants were determined using DQF COSY and 1H NMR experiments. All 2D-NMR data were recorded in the phase-sensitive mode using the time-proportional phase increment method, with 2048 data points in the t2 domain and 512 points in the t1 domain. Slowly exchanging amide protons were identified by lyophilization of a fully protonated sample in H2O solution to dryness, resuspension in 99.99% D_2_O solution and immediate acquisition of a series of 1D spectra. All NMR data were processed using Bruker TOPSPIN 2.1 (Bruker Instruments) or NMRPipe [50] and were analyzed using Sparky 3.12. Solution structures of jS3 were calculated by hybrid distance geometry and simulated annealing protocols in torsion angle space with CNS 1.2 [51]. A total of 656 distance and 22 torsion angle constraints were used for the structure calculations. NOE distance restraints were classified as strong (1.8–3.0 Å), medium (1.8–3.5 Å), weak (1.8–5.0 Å), or very weak (1.8–6.0 Å). Corrections for pseudo atom representations were used for non-stereo specifically assigned methylenes, methyl groups, and aromatic ring protons. Backbone dihedral angle restraints were derived from the 3JHN-Hα coupling constants in the DQF-COSY or 1H NMR spectra in H_2_O solution. MOLMOL [52] and PyMOL [53] were used for structure visualization, and PROCHECK-NMR [54] was used for structure validation. The jasmintide jS3 NMR structure was uploaded to the Protein Data Bank with accession number 5WXE.

### Stability assays

For the thermal stability assay, jS3 were heated in boiling water for 1 h. At each time interval (1.0 h), a 30-μL sample was aliquoted and quenched in an ice bath for 15 min. For the acid stability assay, jS3 and jS4 were incubated in hydrochloric acid (pH 2.0) for 6 h. At each time interval (2, 4 and 6 h), a 30-μL sample was aliquoted and quenched by adding 1 M sodium hydroxide. For the enzymatic stability assay, jS3 and jS4 were incubated for 6 h at the optimal temperature with a buffer solution, as recommended by the manufacturer. At each time interval (2, 4 and 6 h), a 30-μL sample was aliquoted and quenched by adding 1 M hydrochloric acid. For the serum stability assay, jS3 were incubated with 25% human AB-type male serum in phenol red-free Dulbecco’s modified Eagle’s medium at 37 °C for 24 h. At each time interval (4, 8, 12 and 24 h), a 30-μL sample was aliquoted and quenched with 96% ethanol. Samples were kept at 4 °C for 15 min and then centrifuged at 18,000 rpm for 10 min. For all stability assays, the reaction mixture was analyzed by UPLC and monitored by MALDI-MS. The areas under the peak prior to and after treatment were integrated to determine stability. For each stability assay, three independent experiments were performed. At each time point, the sample was quenched by the specific reagent aforementioned and analyzed by UPLC in triplicates.

### Feeding deterrent assay

The antifeedant activity was examined using a no-choice assay [55]. Oat flakes were soaked in different concentrations of jS3 (0.5 to 30 mg/g of oat) and lyophilized. The control diet was prepared in the same manner without addition of jS3. Ten *T. molitor* larvae of similar weights (41 ± 3 mg) were placed in a tissue culture dish with 500 mg of oats. The dishes were transferred into a rearing chamber and kept at 29 ± 1 °C in the dark. After 24 h, the remaining oat flakes were weighed, and the amount consumed was calculated. In total, 30 larvae were used for each concentration, and three independent biological experiments were performed. The feeding deterrence activity was determined as follows [56]:

$$ \mathrm{Feeding}\ \mathrm{Deterrence}\ \mathrm{Index}(FDI)=\left(C-T\right)\ast 100/\left(C+T\right) $$

where C represents the average weight of consumed control diet and T represents the average weight of consumed treatment diet within the groups. The overall differences in feeding amounts were compared to the control and were compared to each other using one-way ANOVA.

### Effect of jasmintides on the growth of larvae

At day 0, 10 *T. molitor* larvae of similar weights (43 ± 2 mg and 47 ± 2 mg in the treatment and control sets, respectively) were fed 200 mg of a diet containing 1 mg of jS3/g of oats. Every three days, the weights of individual larva were recorded, and larvae were transferred to a new dish with the same amount of supplemented diet to maintain a continuous supply. Three independent biological experiments were carried out over a period of 9 days. The control diet was prepared in the same manner without the addition of jS3. Student’s t-test was used to compare the mean with the control.

## Additional files

Additional file 1: **Table S1.** NMR restraints and statistics of the solution structure of jS3. (DOCX 14 kb)

Additional file 2: **Figure S1.** NH/CαH fingerprint region of 2D-NOESY spectrum of jS3 recorded in 90% H_2_O/10% D_2_O at 298 K. Sequential connectivity of each amino acid residue are shown by solid lines. (DOCX 4392 kb)

Additional file 3: **Figure S2.** Summary of sequential and medium NOE connectivity. Slowly exchanging amide protons (filled box) and backbone vicinal coupling constants (open circle: ^3^JHNα < 6 Hz, filled circle: ^3^JHNα > 8 Hz) are indicated. (DOCX 4739 kb)

Additional file 4: **Figure S3.** NOE cross peak between the Hβs of the each disulfide bond in jS3. (DOCX 171 kb)

Additional file 5: **Figure S4.** Annotated MS/MS spectra of jasmintides identified using proteomics approach. Fragments are labeled with *c*-, *z*-, *z* + 1 (*z*’), *z* + 2 (*z*(+ 2)), *b*- and *y*- ions. (DOCX 825 kb)

Additional file 6: **Figure S5.** Phylogenetic tree of the 14 jasmintide precursors. The precursors were aligned using Clustal Omega and the tree was constructed with 1000 bootstraps using neighbor-joining algorithm. (DOCX 17 kb)

Additional file 7: **Table S2.** Comparison of the 14 jasmintides against jS1. (DOCX 12 kb)

Additional file 8: **Figure S6.** The effect of jasmintide jS3 on the cell viability of Sf9 cells. (DOCX 111 kb)

Additional file 9: **Table S3.** TM align score between jasmintide jS3 and other CRPs with 6 cysteine residues. (DOCX 12 kb)

Additional file 10: **Table S4.** The amino acid sequences of jasmintide jS3, human β-defensin hBD1 and conotoxin as24a. (DOCX 12 kb)

## Abbreviations

6C-CRPs: Six-cysteine cysteine-rich peptides
CRPs: Cysteine-rich peptides
D2O: Deuterium oxide
DTT: Dithiothreitol
ETD: Electron-transfer dissociation
FA: Formic acid
FDI: Feeding deterrence index
HPLC: High-performance liquid chromatography
LD_50_: Average lethal dose
MALDI-TOF MS: Matrix-assisted laser desorption/ionization-time of flight mass spectrometry
MS: Mass spectrometry
MS/ MS: Tandem mass spectrometry
NOESY: Nuclear Overhauser effect spectroscopy
PTMs: Post-translation modifications
RMSD: Root-mean-square deviation
RP: Reversed-phase
SCX: Strong cationic exchange
TOCSY: Total correlation spectroscopy
UPLC: Ultra-performance liquid chromatography
