# Supplementary material for: Molecular diversity and function of jasmintides from Jasminum sambac
Source: BMC Plant Biol. 2018 Jul 11;18:144. doi: 10.1186/s12870-018-1361-y (PMC6042386; doi:10.1186/s12870-018-1361-y)
Supplement: Supplementary file 1 — Table S1. NMR restraints and statistics of the solution structure of jS3. (DOCX 14 kb) [file 12870_2018_1361_MOESM1_ESM.docx]

Table S1. NMR restraints and statistics of the solution structure of jS3.

| **Residues** | **NH** | **CαH** | **CβH** | **Others** |
| --- | --- | --- | --- | --- |
| Gln1 | 7.75 | 4.22 | 1.88 | CγH 2.29, 2.39 |
| Leu2 | 8.12 | 4.15 | 1.40,1.35 | CγH 0.87 CδH 0.74, 0.66 |
| Cys3 | 8.25 | 4.52 | 2.99 |  |
| Leu4 | 8.99 | 4.29 | 1.33, 1.17 | CγH 1.09 CδH 0.48, -0.07 |
| Leu5 | 7.62 | 4.38 | 1.58,1.41 | CδH 0.85, 0.88 |
| Cys6 | 7.92 | 4.58 | 3.19, 3.12 |  |
| Gln7 | 9.27 | 4.45 | 2.08 | CγH 2.34, 2.28 NHε^2^ 7.53, 6.76 |
| Thr8 | 7.97 | 4.46 | - | CγH^2^ 1.15 |
| Ser9 | 9.33 | 4.26 | 4.00 |  |
| Arg10 | 7.90 | 4.02 | 1.69 | CγH 1.63, 1.58 CδH 3.11 NHε 7.10 |
| Asp11 | 7.57 | 4.58 | 3.11, 2.83 |  |
| Cys12 | 7.64 | 4.97 | 2.90 |  |
| Asn13 | 7.48 | 4.76 | 2.50, 2.46 | NHδ^2^ 7.35, 6.59 |
| Tyr14 | 7.25 | 3.75 | 3.36,2.81 | CδH 7.28, CεH 6.99 |
| Ile15 | 8.12 | 3.74 | 1.73 | CγH^1^ 1.40, 1.09 CγH^2^ 0.77 CδH^1^ 0.75 |
| Ile16 | 7.89 | 3.68 | 0.82 | CγH^1^ 0.63 CγH^2^ 0.46 CδH^1^ 0.08 |
| Trp17 | 7.16 | 4.44 | 2.99, 2.55 | CδH^1^ 6.94, NεH^1^ 10.27 |
| Thr18 | 7.24 | 4.76 | 4.45 | CγH^2^ 1.09 |
| Val19 | 8.59 | 4.46 | 1.69 | CγH 0.79, 0.71 |
| Cys20 | 8.80 | 4.61 | 2.71, 2.37 |  |
| Arg21 | 8.54 | 4.51 | 1.68, 1.60 | CγH 1.51, 1.34 CδH 3.15, 3.05 NHε 7.64 |
| Asp22 | 9.36 | 4.25 | 3.03, 2.68 |  |
| Gly23 | 7.87 | 4.05, 3.92 | - |  |
| Cys24 | 7.35 | 5.32 | 3.25, 2.58 |  |
| Cys25 | 9.49 | 4.91 | 3.14, 2.61 |  |
| Asn26 | 10.06 | 4.99 | 2.89, 2.53 | NHδ^2^ 7.46, 7.09 |
| Ilel27 | 8.91 | 4.41 | 1.82 | CγH^1^ 1.45, 1.04 CγH^2^ 0.89 CδH^1^ 0.79 |
| Ser28 | 7.95 | 4.14 | 3.85,3.76 |  |
