# Supplementary material for: Molecular diversity and function of jasmintides from Jasminum sambac
Source: BMC Plant Biol. 2018 Jul 11;18:144. doi: 10.1186/s12870-018-1361-y (PMC6042386; doi:10.1186/s12870-018-1361-y)
Supplement: Supplementary file 2 — Figure S1. NH/CαH fingerprint region of 2D-NOESY spectrum of jS3 recorded in 90% H2O/10% D2O at 298 K. Sequential connectivity of each amino acid residue are shown by solid lines. (DOCX 4392 kb) [file 12870_2018_1361_MOESM2_ESM.docx]

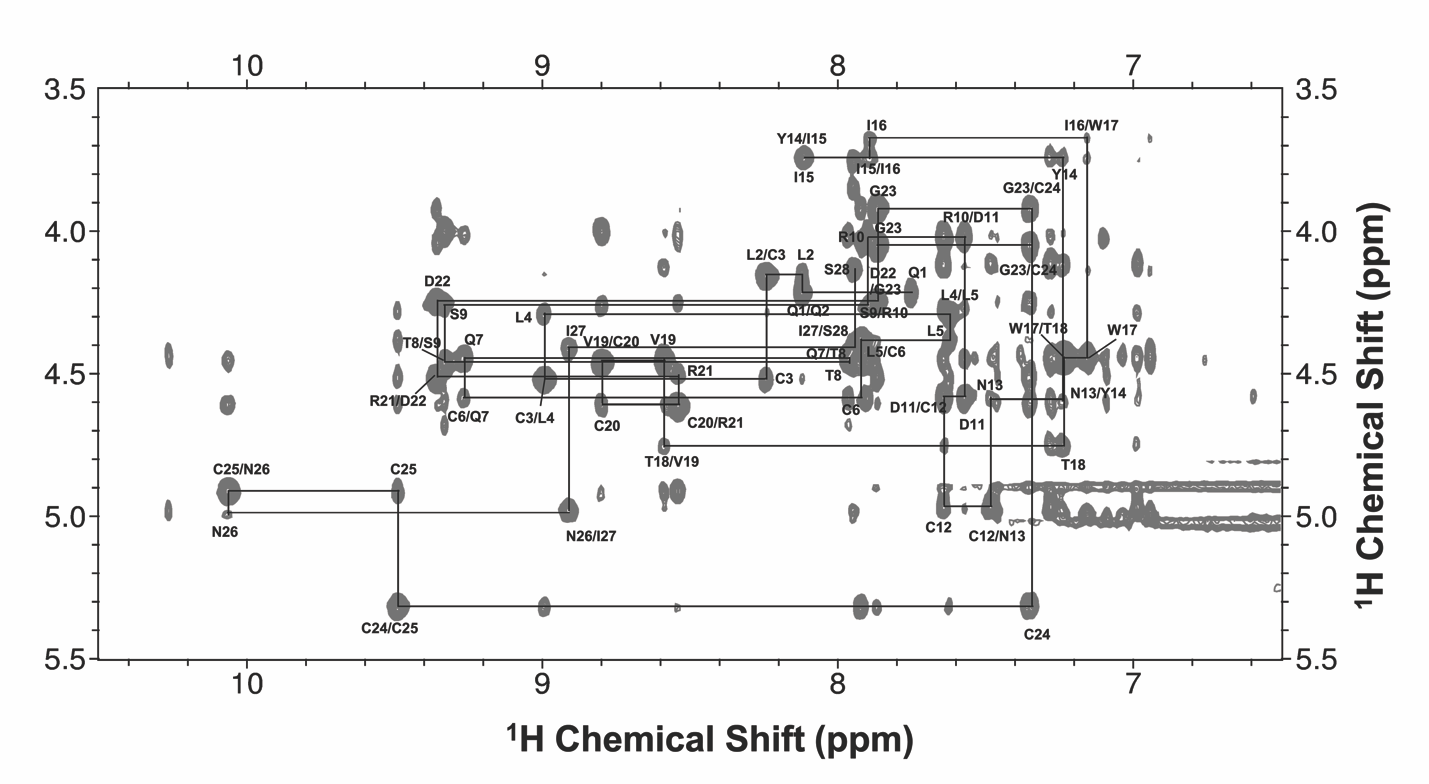


Figure S1. NH/CαH fingerprint region of 2D-NOESY spectrum of jS3 recorded in 90% H2O/10% D2O at 298K. Sequential connectivities of each amino acid residue are shown by solid lines.
