# Supplementary material for: Molecular diversity and function of jasmintides from Jasminum sambac
Source: BMC Plant Biol. 2018 Jul 11;18:144. doi: 10.1186/s12870-018-1361-y (PMC6042386; doi:10.1186/s12870-018-1361-y)
Supplement: Supplementary file 3 — Figure S2. Summary of sequential and medium NOE connectivity. Slowly exchanging amide protons (filled box) and backbone vicinal coupling constants (open circle: 3JHNα < 6 Hz, filled circle: 3JHNα > 8 Hz) are indicated. (DOCX 4739 kb) [file 12870_2018_1361_MOESM3_ESM.docx]

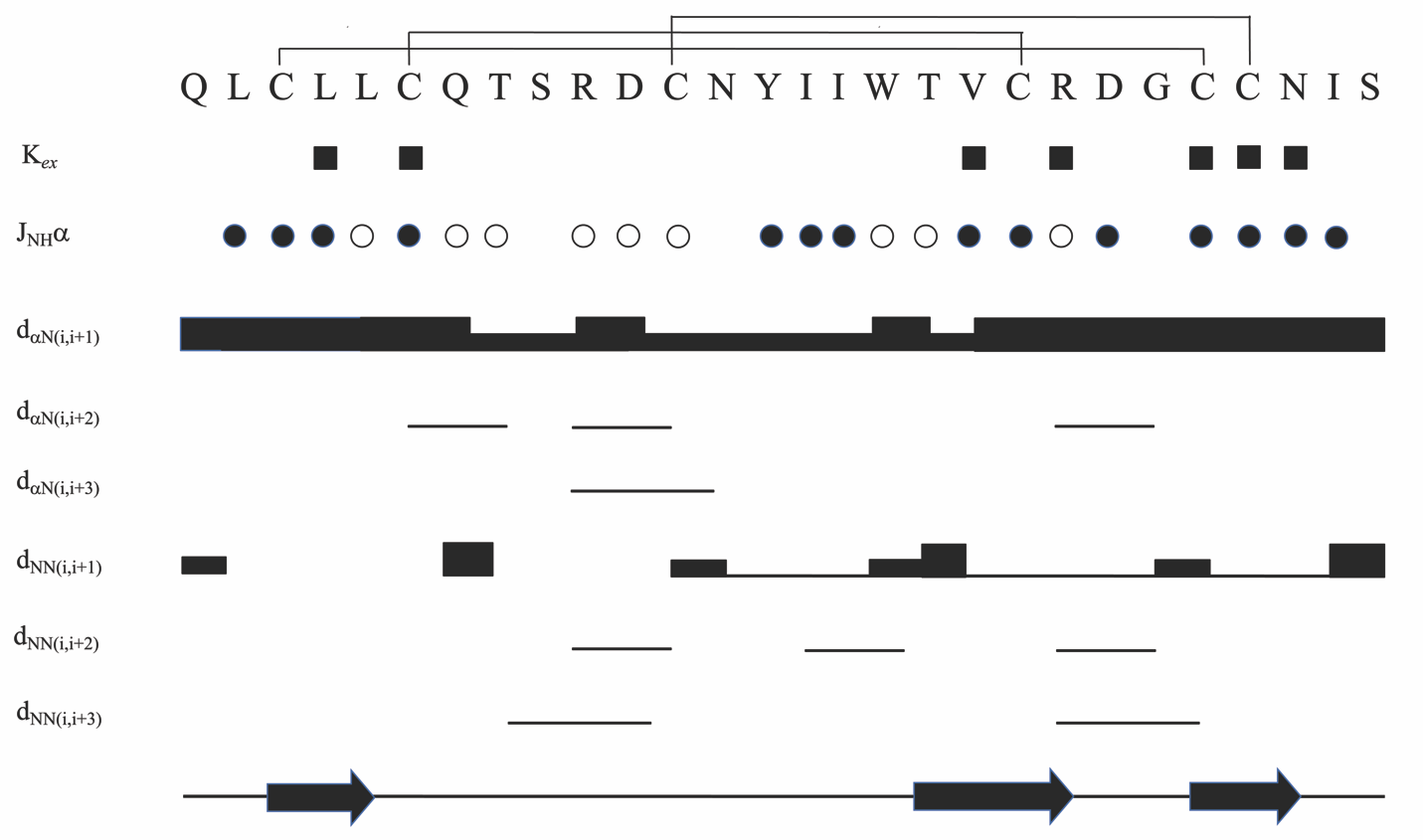


Figure S2. Summary of sequential and medium NOE connectivity. Slowly exchanging amide protons (filled box) and backbone vicinal coupling constants (open circle: ^3^JHNα < 6Hz, filled circle: ^3^JHNα > 8Hz) are indicated.
