# Supplementary figures and images for: Molecular diversity and function of jasmintides from Jasminum sambac
Source: BMC Plant Biol. 2018 Jul 11;18:144. doi: 10.1186/s12870-018-1361-y (PMC6042386; doi:10.1186/s12870-018-1361-y)

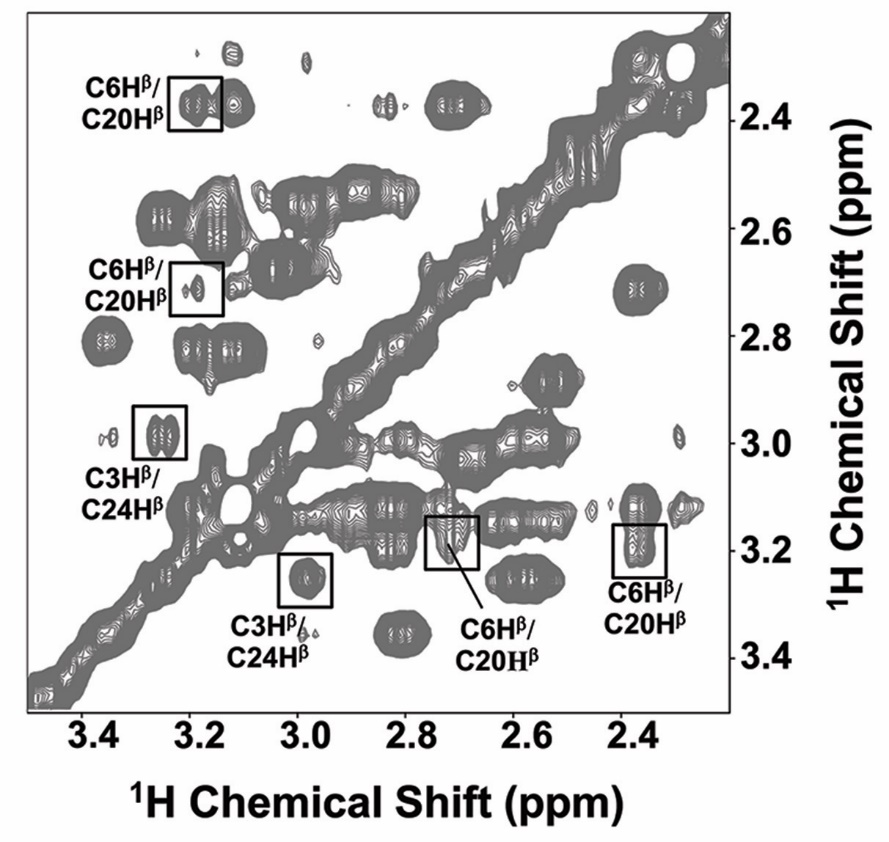


Figure S3. NOE cross peak between the Hβs of the each disulfide bond in jS3.

Supplement: Supplementary file 4 — Figure S3. NOE cross peak between the Hβs of the each disulfide bond in jS3. (DOCX 171 kb) [file 12870_2018_1361_MOESM4_ESM.docx]

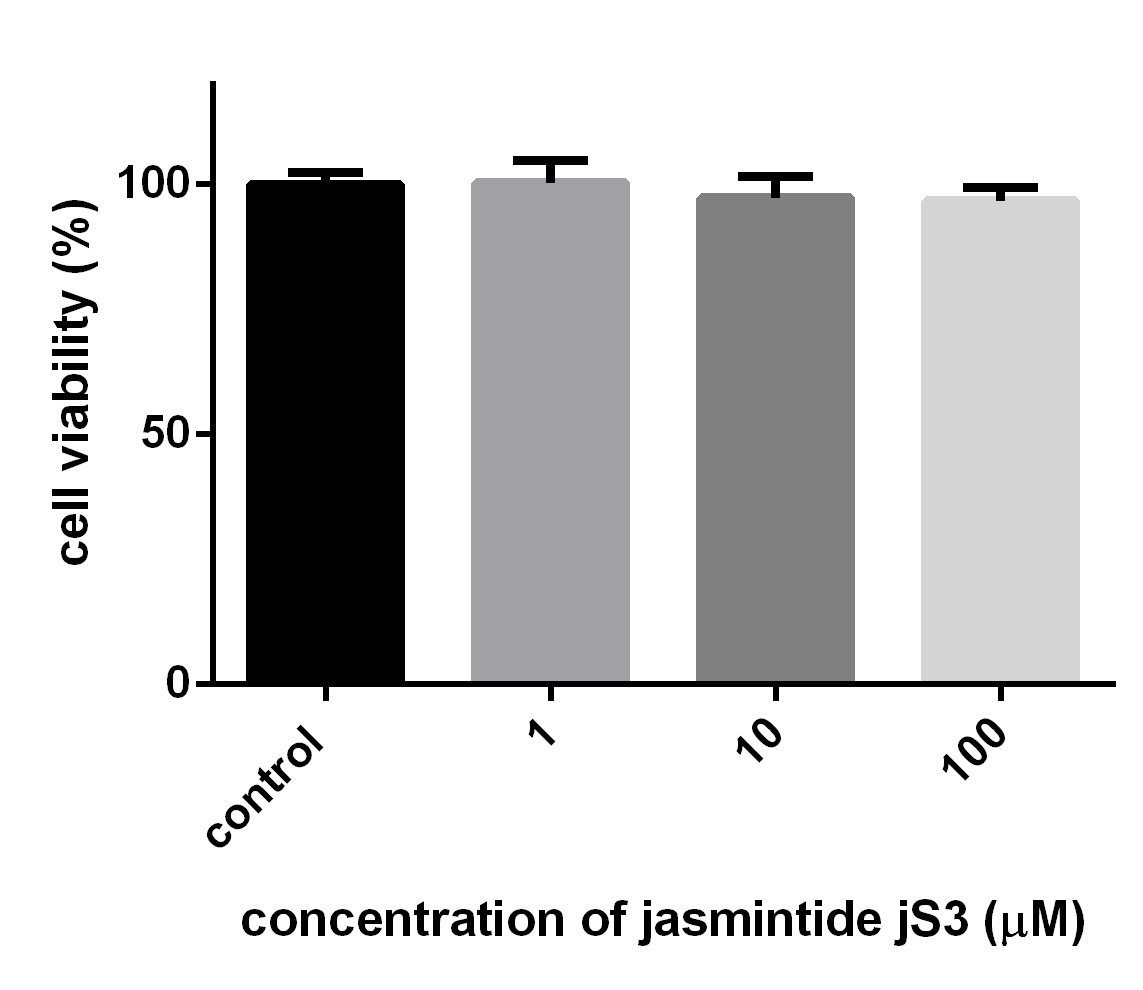


Figure S6. The effect of jasmintide jS3 on the cell viability of Sf9 cells.

Supplement: Supplementary file 8 — Figure S6. The effect of jasmintide jS3 on the cell viability of Sf9 cells. (DOCX 111 kb) [file 12870_2018_1361_MOESM8_ESM.docx]
