# Supplementary material for: Molecular diversity and function of jasmintides from Jasminum sambac
Source: BMC Plant Biol. 2018 Jul 11;18:144. doi: 10.1186/s12870-018-1361-y (PMC6042386; doi:10.1186/s12870-018-1361-y)
Supplement: Supplementary file 5 — Figure S4. Annotated MS/MS spectra of jasmintides identified using proteomics approach. Fragments are labeled with c-, z-, z + 1 (z’), z + 2 (z(+ 2)), b- and y- ions. (DOCX 825 kb) [file 12870_2018_1361_MOESM5_ESM.docx]

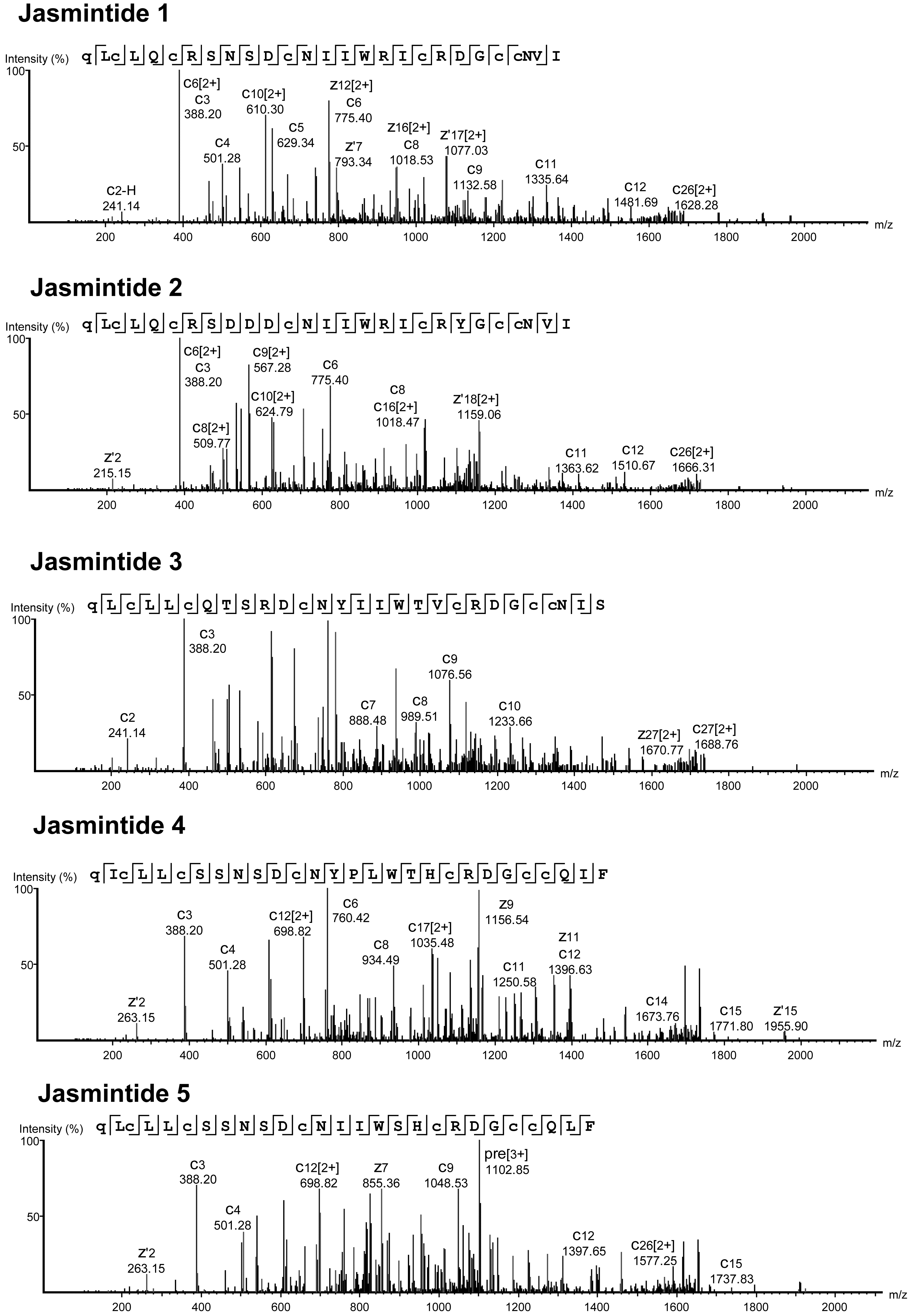


**
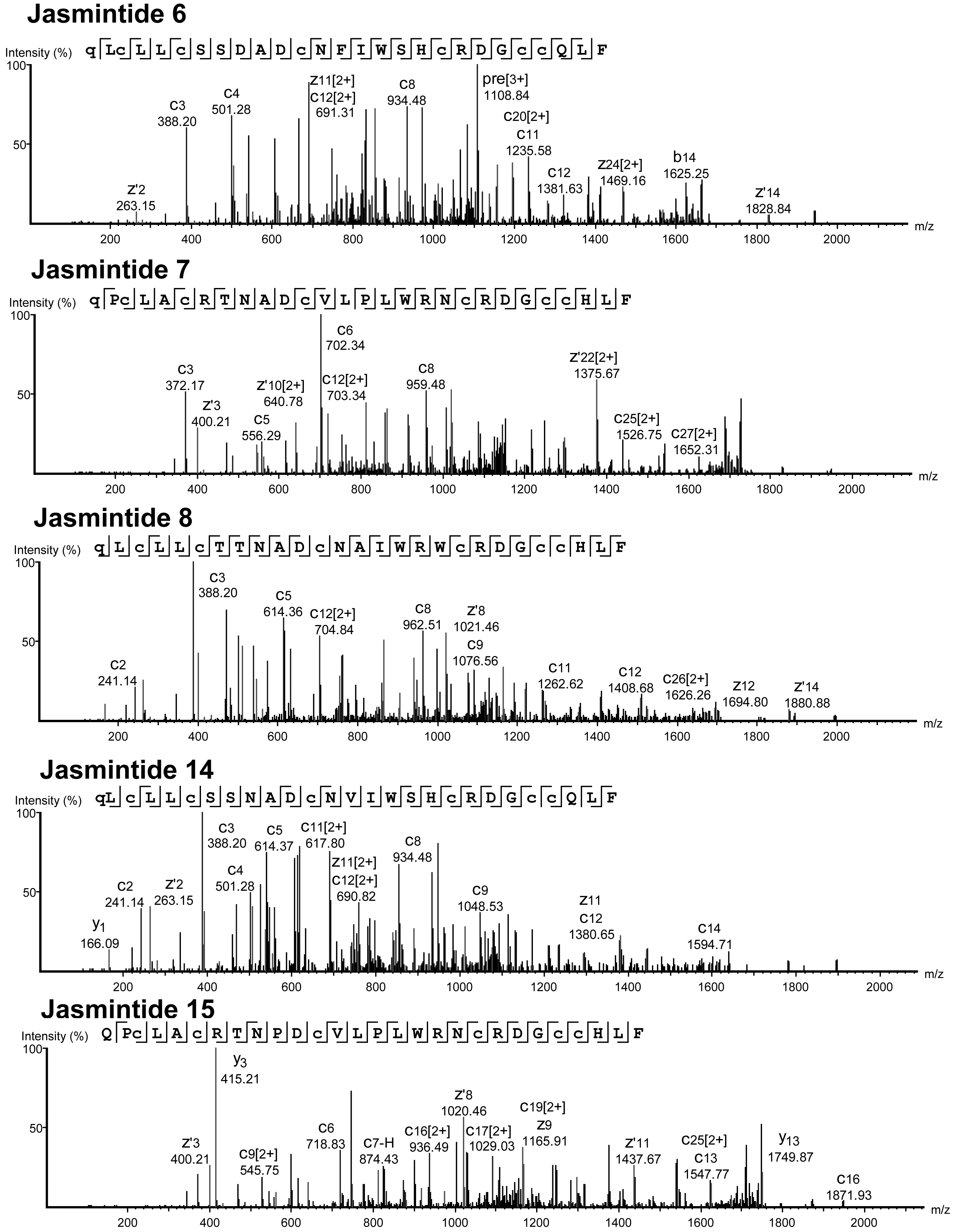
**

Figure 4. Annotated MS/MS spectra of jasmintides identified using proteomics approach. Fragments are labeled with *c*-, *z*-, *z*+1 (*z*’), *z*+2 (*z*(+2)), *b*- and *y*- ions.
