# Supplementary material for: Molecular diversity and function of jasmintides from Jasminum sambac
Source: BMC Plant Biol. 2018 Jul 11;18:144. doi: 10.1186/s12870-018-1361-y (PMC6042386; doi:10.1186/s12870-018-1361-y)
Supplement: Supplementary file 6 — Figure S5. Phylogenetic tree of the 14 jasmintide precursors. The precursors were aligned using Clustal Omega and the tree was constructed with 1000 bootstraps using neighbor-joining algorithm. (DOCX 17 kb) [file 12870_2018_1361_MOESM6_ESM.docx]

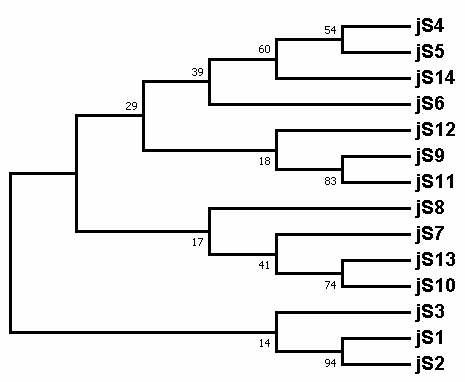


Figure S5. Phylogenetic tree of the 14 jasmintide precursors. The precursors were aligned using Clustal Omega and the tree was constructed with 1000 bootstraps using neighbor-joining algorithm.
