# Supplementary material for: Molecular diversity and function of jasmintides from Jasminum sambac
Source: BMC Plant Biol. 2018 Jul 11;18:144. doi: 10.1186/s12870-018-1361-y (PMC6042386; doi:10.1186/s12870-018-1361-y)
Supplement: Supplementary file 7 — Table S2. Comparison of the 14 jasmintides against jS1. (DOCX 12 kb) [file 12870_2018_1361_MOESM7_ESM.docx]

Table S2. Comparison of the 14 jasmintides against jS1.

| Jasmintides | Identity (%) | Similarity (%) |
| --- | --- | --- |
| jS2 | 88.9 | 92.6 |
| jS3 | 66.7 | 85.2 |
| jS4 | 63 | 74.1 |
| jS5 | 76.9 | 80.8 |
| jS6 | 65.4 | 76.9 |
| jS7 | 59.3 | 81.5 |
| jS8 | 69.2 | 84.6 |
| jS9 | 48 | 68 |
| jS10 | 40 | 65 |
| jS11 | 48 | 72 |
| jS12 | 27.3 | 36.4 |
| jS13 | 54.5 | 59.1 |
| jS14 | 69.2 | 80 |
| jS15 | 59.3 | 77.8 |

The sequences were aligned and compared using EMBOSS Water.
