# Supplementary material for: Molecular diversity and function of jasmintides from Jasminum sambac
Source: BMC Plant Biol. 2018 Jul 11;18:144. doi: 10.1186/s12870-018-1361-y (PMC6042386; doi:10.1186/s12870-018-1361-y)
Supplement: Supplementary file 9 — Table S3. TM align score between jasmintide jS3 and other CRPs with 6 cysteine residues. (DOCX 12 kb) [file 12870_2018_1361_MOESM9_ESM.docx]

Table S3. TM align score between jasmintide jS3 and other CRPs with 6 cysteine residues.

| **CRP family** | **Peptide** | **PDB** | **TM align score** |
| --- | --- | --- | --- |
| Jasmintide | jS1 | 2N5Q | 0.55955 |
| β-Ginkgotide | β-gB1 | 5XIV | 0.21402 |
| CKAI | AAI | 1QFD | 0.23020 |
|  | aL1 | 2MI9 | 0.28167 |
|  | wR1 | 2MAU | 0.26515 |
| Carboxypeptidase inhibitor | PCI | 1H20 | 0.42022 |
| 6C-Hevein-like peptide | aSG1 | N.A. | 0.40015 |
|  | Ac-AMP | 1MMC | 0.38430 |
|  | Sm-AMP-1 | 2KUS | 0.35153 |
|  | Sm-AMP-2 | 2N1S | 0.38002 |
| Thionin | Crambin | 3U7T | 0.24526 |
|  | gamma 1H | 1GPT | 0.32069 |
|  | hellethionin D | 3SZS | 0.24686 |
|  | NaD1 | 1MR4 | 0.31810 |
|  | Viscotoxin B | 1JMP | 0.21572 |
| Human defensin | hBD1 | 1KJ5 | 0.32582 |

Pairwise alignment using jasmintide jS3 was performed. N.A.: Not available. The structure of aSG1 was kindly provided by the authors. A TM-score value <0.3 means that the two candidates have random structural similarity, whereas a value >0.5 means that they shared the same protein fold.
