# Supplementary material for: Molecular diversity and function of jasmintides from Jasminum sambac
Source: BMC Plant Biol. 2018 Jul 11;18:144. doi: 10.1186/s12870-018-1361-y (PMC6042386; doi:10.1186/s12870-018-1361-y)
Supplement: Supplementary file 10 — Table S4. The amino acid sequences of jasmintide jS3, human β-defensin hBD1 and conotoxin as24a. (DOCX 12 kb) [file 12870_2018_1361_MOESM10_ESM.docx]

Table S4. The amino acid sequences of jasmintide jS3, human β-defensin hBD1 and conotoxin as24a.

| Peptide | Amino acid sequence |
| --- | --- |
| jS3 | --------QLCLL----CQTSRDCNYI--IWTVCRDG---CCNIS-- |
| hBD1 | GLGHRSDHYNCVSSGGQCLYSA-CPIFTKIQGTCYRGKAKCCK---- |
| as24a | ----------CK-----C-PS--CNFND-VTENC---K--CCIFRQP |
